# Uncommon cause of liver abscess

**DOI:** 10.1002/ccr3.1691

**Published:** 2018-07-01

**Authors:** Andre R. Dias, Daniel J. Szor, Claudia B. A. Ferreira, Carmen L. Navarro

**Affiliations:** ^1^ Hospital Santa Cecilia São Paulo Brazil

**Keywords:** bowel perforation, fishbone, foreign body, hepatic abscess

## Abstract

Gastrointestinal perforation by fishbone causing a liver abscess is a rare entity, but should be included in the differential diagnosis to avoid delay in the treatment.

## 1 CASE PRESENTATION

This 35‐year‐old male patient had accidentally swallowed a fishbone 2 months prior to his admittance in the emergency room. At that time, he presented pain in the upper abdomen a few days after ingestion.

**Figure 1:**
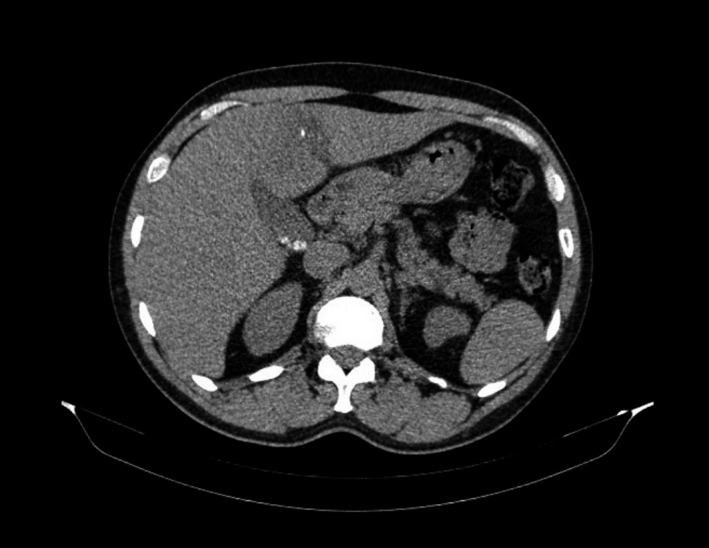
Computed tomography axial image showing a linear calcified body inside the liver abscess

**Figure 2:**
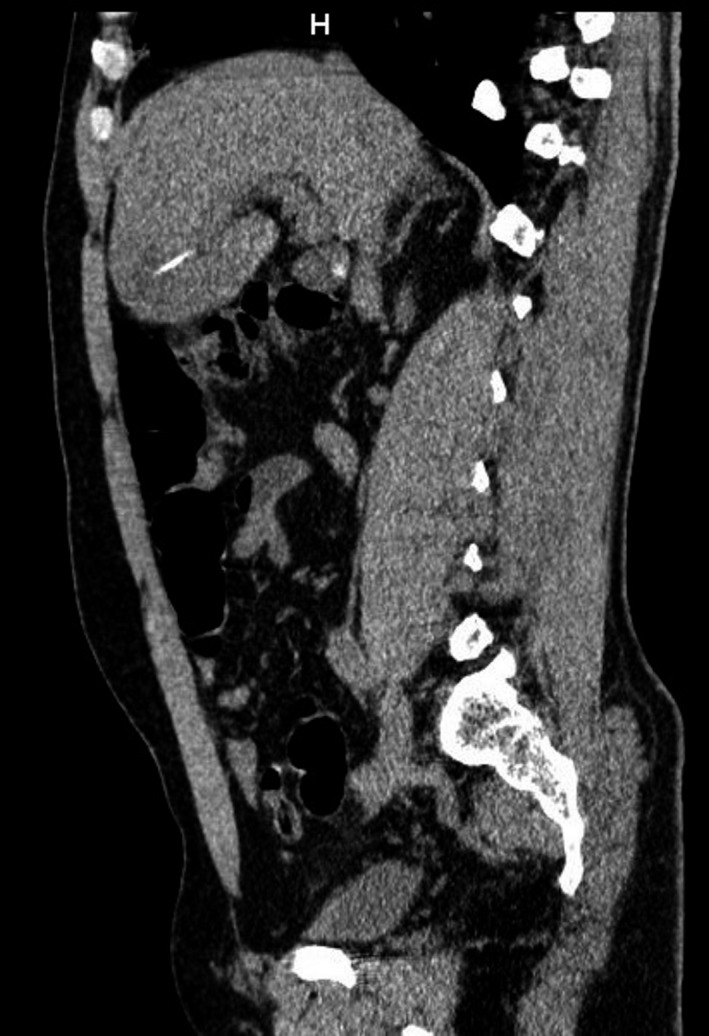
Computed tomography sagital image showing a linear calcified body inside the liver abscess

**Figure 3:**
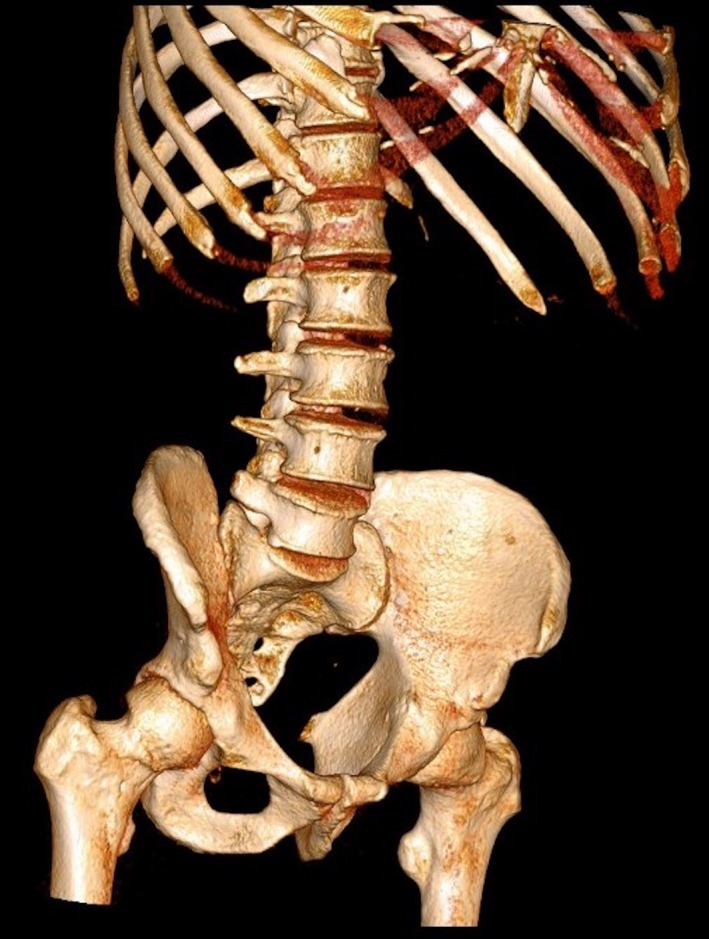
Tomographic 3D reconstruction showing a linear calcified body in liver topography

**Figure 4:**
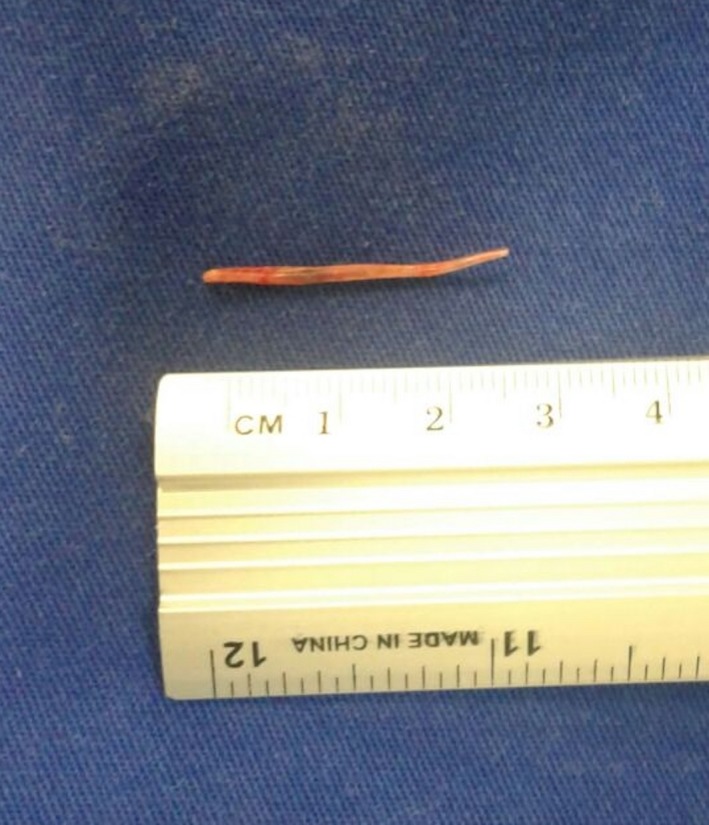
Surgical specimen

He presented abdominal pain and fever. Computed tomography scan showed a liver abscess with a linear calcified body inside (Figures 1, 2, 3). Due to the symptoms, a laparotomy was indicated. Transverse colon was intimately adhered to the liver; after freeing the adhesion, no colic perforation was observed. The abscess was drained and the fishbone removed (Figure 4). Postoperative period was uneventful.

The diagnosis of a liver abscess caused by fishbone gastrointestinal perforation is difficult, due to its rare nature and also because it is difficult for the patient to remember the accidental ingestion.^1^ In these cases, complementar imaging exams are fundamental to find a calcified foreign body inside the abscess. Minimally invasive approaches, although not adopted in this particular case, are feasible and described in literature.^2^

## CONFLICT OF INTEREST

None declared.

## AUTHORSHIP

ARD: prepared the manuscript, member of surgical team; DJS: prepared the manuscript, member of surgical team; CBAF: reviewed the article; CLN: reviewed the article.
